# Impact of Sample Size and Deformation Measurement Techniques on Uniaxial Tensile Testing of Fiber-Based Materials

**DOI:** 10.3390/ma19061197

**Published:** 2026-03-18

**Authors:** Yuchen Leng, Cedric W. Sanjon, Peter Groche, Marek Hauptmann, Jens-Peter Majschak

**Affiliations:** 1 TU Darmstadt Institute for Production Engineering and Forming Machines, 64287 Darmstadt, Germany; 2 Fraunhofer Institute for Process Engineering and Packaging IVV, 01189 Dresden, Germany; marek.hauptmann@ivv-dd.fraunhofer.de (M.H.);; 3 TU Dresden Institute for Processing Machines and Processing Technology, 01062 Dresden, Germany

**Keywords:** paper, paperboard, stress–strain curve, size-effect, DIC, inhomogeneity

## Abstract

The uniaxial tensile test is a common and fundamental test in materials science and engineering, in which a specimen is subjected to controlled tension until failure. From this, the stress–strain curve and many property parameters of the material can be calculated, such as tensile strength, ultimate strength, maximum elongation, Young’s modulus, Poisson’s ratio, and yield strength. As fibrous materials, such as paper and paperboard, become more popular, accurately measuring their mechanical properties becomes essential for developing and applying these materials, especially in packaging. However, since they are anisotropic and inherently inhomogeneous due to the arrangement of the fibers, accurately determining their mechanical properties is not straightforward. This study investigated how several key factors influence the results of tensile tests on fiber-based materials: sample size and deformation measurement techniques using three fiber materials. This study also compared three different strain recording methods: digital image correlation (DIC), video extensometer, and conventional extensometer (Traverse). The DIC technique emphasized the effect of the inherent inhomogeneity of the paperboard on the overall mechanical properties obtained from tensile tests. The results indicated that sample size has a negligible effect on the stress–strain curve, and any apparent influence likely stems from slip at the grips during tensile testing. However, sample size does affect paperboard fracture to some extent. The study also provided recommendations for optimal specimen geometry and deformation recording methods to improve the accuracy and repeatability of tensile testing of fiber-based materials.

## 1. Introduction

Paper and paperboard consist of a network of cellulose fibers that are bonded together through hydrogen bonds and mechanical entanglement. These fibers originate from different sources and exhibit varying average lengths, including softwoods which can reach up to 3.6 mm, and hardwoods which can reach up to 1.2 mm [1]. The structure of the fiber network, including the distribution of fiber length, the bonding area, and the network topology, fundamentally controls the macroscopic mechanical properties of paper materials [2]. Paper and paperboard are generally considered to be an anisotropic and inhomogeneous material due to the disposition of fiber suspensions in the paper machine and the nonuniform distribution of fiber orientations during production. Tensile stiffness and strength are higher in the machine direction (MD), where the majority of fibers are aligned, compared to the cross direction (CD), while the thickness direction has the fewest fibers. The fiber orientation distribution can be approximated by probability density functions, which are directly linked to the anisotropic stiffness and strength characteristics observed in tensile testing [3]. Tensile stiffness and strength are higher in the machine direction (MD) than in the cross direction (CD), where the majority of fibers are aligned. The thickness direction (ZD) has the fewest fibers. Thus, characterizing the material properties of paper and paperboard is not simple, and the required in-plane and out-of-plane experiments are described in the authors’ previous review article [4]. The authors previously proposed also a transmitted light method to determine cardboard irregularities, including grammage, thickness, and fiber orientation [5].

The uniaxial tensile test is the most commonly used method for determining the in-plane mechanical properties of paper and paperboard, such as Young’s modulus, yield strength, tensile strength, and elongation at break [2,6]. A tensile testing machine generally consists of a fixed traverse and a moving traverse driven electrically or hydraulically by a spindle. The traverse moves in one direction at a defined speed, depending on the test specification, to tear the tensile specimen, which is clamped between the crossheads by specimen clamps. Traditionally, the deformation of the specimen is recorded by the traverse’s travel. However, the traditional experimental method has two drawbacks. One is that tensile strength cannot be accurately measured because stress is concentrated at the clamp, causing failure at the clamp. Second, tensile strain is measured by the amount of elongation between the clamps, i.e., traverse movement. Nevertheless, strain can also include the effects of machine compliance, such as fixture backlash and deformation, and sliding between the specimen and fixture [7]. When testing highly ductile, highly elastic, contact-sensitive, or specialized specimens, a non-contact optical measurement system enables to measure strain accurately. In addition to the traditional traverse method, which can introduce errors due to slippage and specimen damage at the attachment points, two other non-contact strain measurement methods are video extensometer and Digital Image Correlation (DIC). A video extensometer uses video technology to track reference marks applied to the material and measure the strain of a sample. It offers a high level of precision, but it is sensitive to lighting conditions and the surface quality of the specimen [8]. DIC is another non-contact optical method that tracks the deformation of a material by comparing digital images taken before and during loading. This technique provides full-field strain measurements with greater accuracy (below 5 microstrain) and spatial resolution (around 100 microstrain) than strain gauges, making it ideal for heterogeneous and anisotropic materials, such as fiber-based products [9,10]. Advanced applications of DIC, when combined with inverse methods such as the Virtual Fields Method (VFM), have enabled the identification of smoothly varying in-plane stiffness heterogeneity [11,12].

The commonly used paper testing standards, ISO 1924 [13], TAPPI T494 [14], and ASTM D828 [15], measure the tensile strength of paper products. Accordingly, it is recommended to utilize a sample width of either 15 or 25 mm, a gauge length of 180 mm (i.e., the distance between the clamps), and a constant traverse speed of 20 to 25 mm/min. Due to its inherent inhomogeneity and significantly greater randomness, paper exhibits lower repeatability than metallic materials in tensile testing. Local variations in basis weight, fiber orientation, thickness, and density create weak spots within the paper structure that strongly influence local strain distribution and failure initiation during mechanical loading [16,17]. Statistical analyses have shown that local grammage is the strongest predictor of local bond failure, while fiber alignment in the loading direction correlates with reduced local strain [16]. These microstructural heterogeneities at scales comparable to fiber flocs produce nonuniform, nonaffine deformations and strain hot spots that govern tearing mechanisms and contribute to specimen-to-specimen variability in tensile test results [18]. Some studies have investigated the factors contributing to significant deviations in the results of tensile testing of paper with varying sample sizes. Using paper with different strip lengths revealed that as the strip length increases, the yield strength and ductility decrease (lower strain at break), while Young’s modulus increases [19]. Using two sample sizes, it was found that the failure strain doubled as the width-to-length ratio increased from 0.3 to 3 [20]. However, since only two geometries were used, no definitive conclusion can be reached, especially since the small specimens showed a high level of error. Analytical and numerical findings demonstrated that the tensile stress–strain behavior is highly sensitive to specimen size. Specifically, larger specimens exhibit a significant decrease in tensile strength and an increase in strain at break in the CD (cross direction) relative to the MD (machine direction) [21]. Recently, stress–strain curves were compared for three materials with varying widths and lengths, as well as strain at break versus length-to-width ratio. Hagman and Nygards [22] found that the presence of strain streaks within the sample results in a size effect concerning the length-to-width ratio. This effect can be attributed to the activation of strain zones within the sample. This effect can be attributed to the activation of strain zones within the sample. Additional research on zero-span testing has shown that fiber orientation within the grip region can lead to deviations from theoretical predictions, potentially overestimating individual fiber strength [23]. Furthermore, specimen geometry, including the presence of notches and the aspect ratio, significantly affects failure mechanisms: MD specimens tend to fail abruptly with angular fracture paths, while CD specimens exhibit internal delamination [24]. These geometry- and direction-dependent failure modes underscore the importance of carefully selecting specimen dimensions and orientations when characterizing paper and paperboard materials.

Accurate stress–strain curves are also critical for understanding material behavior and developing accurate material models for numerical simulations. Parameters such as tensile strength and strain at break significantly impact damage modeling and processing simulation [4]. Because the uniaxial tensile test is the most fundamental method of characterizing materials, it is necessary to have a deep understanding of all the factors influencing the experiments, such as test methods, sample size, and sample direction. This study investigated how specimen size and deformation recording methods influence tensile test results. Paperboard samples of different lengths and widths were used for tensile testing, and the effect of different tensile speeds on the mechanical response was examined. The differences in results between the conventional traverse method, the non-contact video extensometer method, and the digital image correlation (DIC) full-field measurement technique were compared. Influencing factors were analyzed, and recommendations were made for tensile testing of paper and paperboard as fiber-based materials.

## 2. Materials and Methods

### 2.1. Materials

The selection of sample materials covered specific fiber types and production processes. Three different uncoated paperboards from three different producers were investigated. Paperboard A (Enviro, Inapa, Sintra, Portugal) is a premium recycled paper made from 100% recycled fibers. It is commonly used for printing purposes. Paperboard B (Trayforma, Stora Enso, Helsinki, Finland) is a three-layer fiber construction board with chemithermomechanical pulp (CTMP) in the middle layer, providing it with excellent tensile strength. Paperboard C (Fibreform, KAPAG, Muhen, Switzland) is a paperboard with excellent stretchability and almost isotropic properties. Paperboard A consists of recycled fibers, while paperboards B and C consist of virgin fibers. However, due to differences in production technology, paperboards B and C exhibit different isotropic properties. Detailed parameters are shown in Table 1. The MD-CD ratio refers to the ratio of mechanical or physical properties measured in the machine direction (MD) to those measured in the cross direction (CD) for paper or paperboard, and it is commonly used to characterize anisotropy resulting from fiber orientation during manufacturing. In this table, it refers to the tensile stiffness ratio between MD and CD.

### 2.2. Test Configuration

The paperboards were tested in three directions, namely MD, CD, and a 45° direction. As for the geometry, the gauge length varied from 30, 60, 90 to 120 mm, while the sample width varied from 30, 15 to 5 mm. All specimens were cut using a vacuum cutting plotter to ensure geometric accuracy.

**Figure 1:**
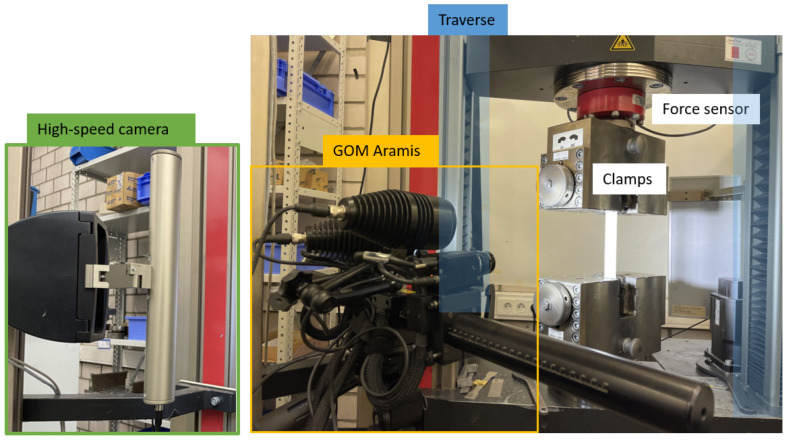
Illustration of test setup with traverse, video extensometer and GOM Aramis.

In order to compare the difference of the deformation recording methods and their influence on the measurement of the mechanical properties of fiber-based materials, the traditional traverse (TRA), video extensometer (VID), and DIC methods were applied. The Zwick/Roell Z100 material testing machine (ZwickRoell Group, Ulm, Germany) incorporated with three deformation recording methods (see Figure 1): (1) build-in traverse; (2) with high-resolution video extensometer; (3) with GOM Aramis as 3D deformation measurement. The recording frequency was 12 Hz for materials A and B, and 6 Hz for material C due to the extended duration of the experiment.

Tensile tests were conducted in a room conditioned at standard temperature 23 °C and 42% relative humidity, with a constant tensile speed of 20 mm/min. A total of 5 replications were performed for each experimental matrix and for subsequent analysis.

### 2.3. Sample Preparation

**Figure 2:**
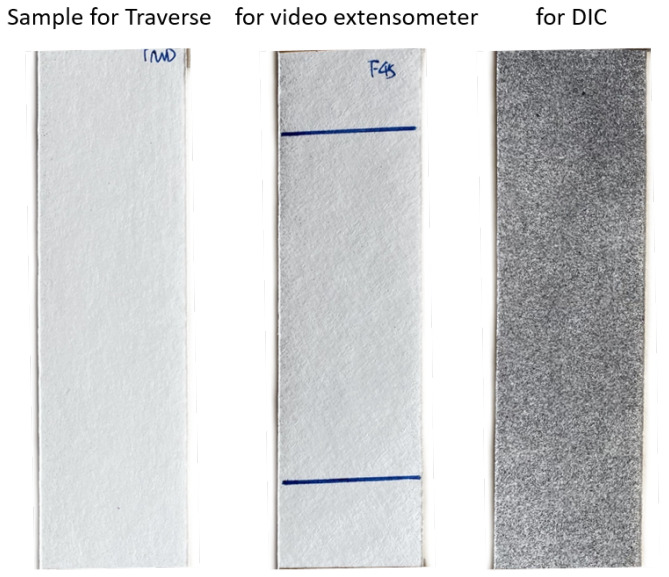
Comparison of sample preparation for 3 test setups: Traverse, Video extensometer with marked lines, and DIC with sprayed pattern.

Due to the differences in the test setups, different specimen preparation methods were required, as shown in Figure 2. For setup 1, which used the traverse as the deformation recording tool, no special marking on the specimen in the tensile test was required. For setup 2, using the video extensometer as the deformation recording tool, two parallel, slightly inclined lines were inscribed on the specimens as high-speed camera tracking targets. For the latest DIC method using the GOM Aramis setup as the deformation recording tool, the specimen surface was carefully sprayed with random black spots to serve as references for the DIC analysis.

## 3. Results

### 3.1. Comparison of Three Materials and Three Deformation Recording Methods

**Figure 3:**
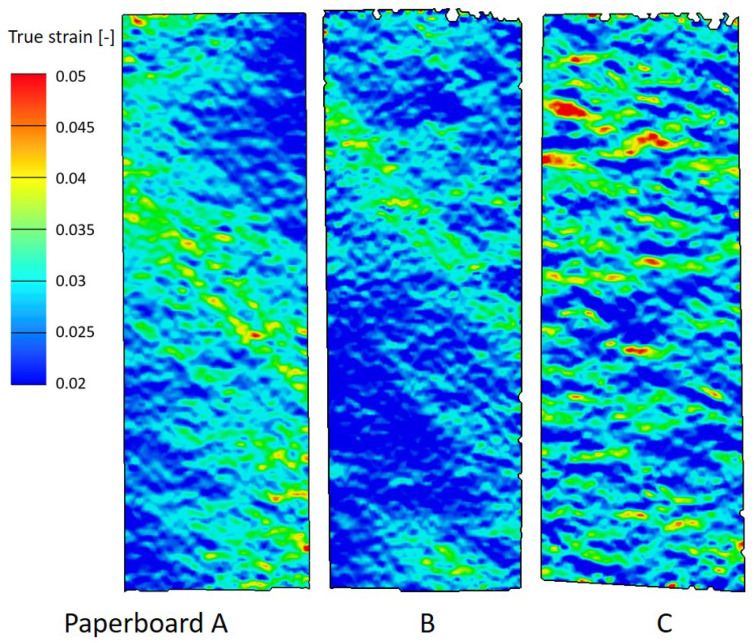
True strain distribution of 45°-samples with sample size 90 × 30 mm^2^ from paperboard A, B, and C.

Figure 3 illustrated the true strain distribution from DIC measurement of 45° samples from three types of paperboard under nearly the same tensile force. Diagonal striations were visible on samples A and B, aligning with their primary fiber orientation. However, this feature was not observable on Paperboard C, which has an almost isotropic property.

**Figure 4:**
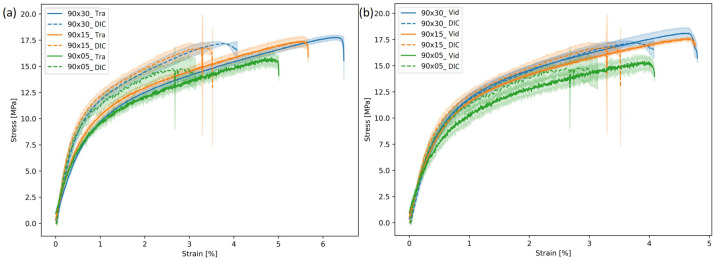
Comparison of stress–strain curves of (**a**) traverse (Tra) and DIC method; (**b**) video extensometer (Vid) and DIC method for material A in CD with sample size 90 × 30 mm^2^; shaded areas represent deviation.

Figure 4 illustrated the comparison of the true stress–strain curves using the traverse versus DIC and video extensometer versus DIC, based on five repeated tests (subsequent analyses likewise). The areas with lower transparency represented deviations in the curve across five repetitions; this applied to all subsequent figures. To ensure consistent evaluation lengths, the three methods were not compared side by side. The test series was conducted under identical conditions, with the same geometry, material, direction, and tensile speed. The strain results in the DIC stress–strain curve were derived from the average value across the entire strain field of the sample. The results from Traverse and DIC showed significant differences, with Traverse’s curve clearly skewed to the right, while the video extensometer and DIC results were closely aligned.

According to Figure 2, the differences in the principles of these three test setups resulted in discrepancies in the results. The traditional traverse method only measures deformation of the specimen’s gripping section, i.e., the top and bottom edges. However, despite the specimen’s low fracture strength, the stiffness of the testing equipment inevitably affected the experimental results. Furthermore, the residual stress of the specimen may impact the precise measurement of force and displacement, particularly for shorter, wider specimens, where clamping may generate pre-stress. In addition to pure specimen elongation, the measured elongation also included deformation and displacement outside the specimen area (e.g., prestressing, fixture deformation, machine clearance), whereas the other two methods only recorded deformation occurring within the evaluated region of the specimen. Additionally, since displacement was measured in contact, minor slippage of the specimen could affect the overall result. A separate video extensometer equipped with a high-speed camera captures changes in the distance between two parallel measuring marks. This device operates independently of the tensile testing machine to ensure that any machine deformation does not affect specimen deformation measurements. GOM Aramis uses triangulation to provide precise, full-field, and point-based 3D coordinates for measurements, enabling real-time recording of the entire specimen’s deformation. However, this method has limitations, such as measurement noise (especially for strains below 0.05%). There exists a 0.5–1% elongation deviation between these two methods, as the DIC focuses on whole-field strain. After arithmetic averaging, the resulting strain value was smaller than that calculated by the video extensometer based on two marker lines. It was evident that the video extensometer and GOM Aramis demonstrated higher accuracy when measuring strain in tensile testing of paperboard materials, and GOM Aramis is particularly suited to measuring local deformation in inhomogeneous materials.

### 3.2. Effect of Geometric Size on Mechanical Properties

Table 2 summarized the effect of geometry, especially length (L) and width (W), on the stress–strain curve, maximum stress, and maximum strain using these three measurement methods. To evaluate correlations, the consistency of the trends observed in all relevant experiments was analyzed. A value of 1 was assigned when a clear correlation was observed in all tests, and a value of 0 was assigned when a correlation was weak or inconsistent. The assessment was rather based on the qualitative agreement of the trends in the experimental data.

As shown in the table, the correlation between the geometric dimensions of the sample and the stress–strain curve was significantly higher for the traverse method than for the other two methods. However, the correlation remained non-significant, with a sum of 10 with all three methods. The influence of geometric dimensions on fracture properties was more significant, with sums of 33 and 34 for maximum stress and maximum strain, respectively, with all three methods. There was no discernible difference in the impact of specimen length and width on measured mechanical properties. This is the combined effect of the internal fiber arrangement within the paperboard, internal stresses and slip, as well as measurement errors.

#### 3.2.1. Effect of Specimen Length

**Figure 5:**
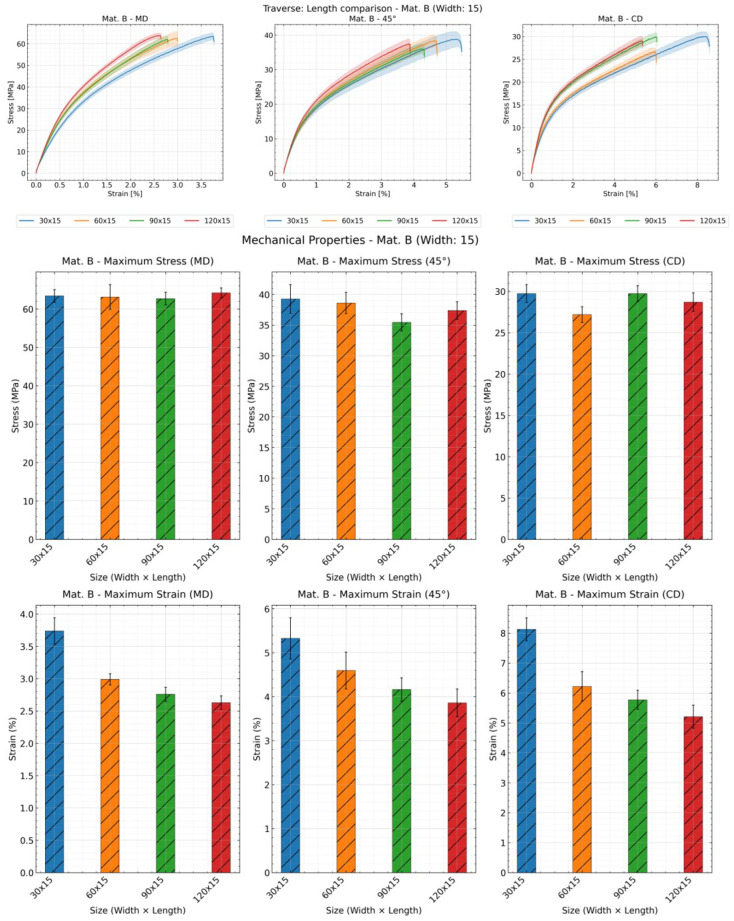
Effect of specimen length on the mechanical properties: stress–strain curve, tensile strength and strain at break of material B with sample width of 15 mm and all lengths using traverse; shaded areas represent deviation.

**Figure 6:**
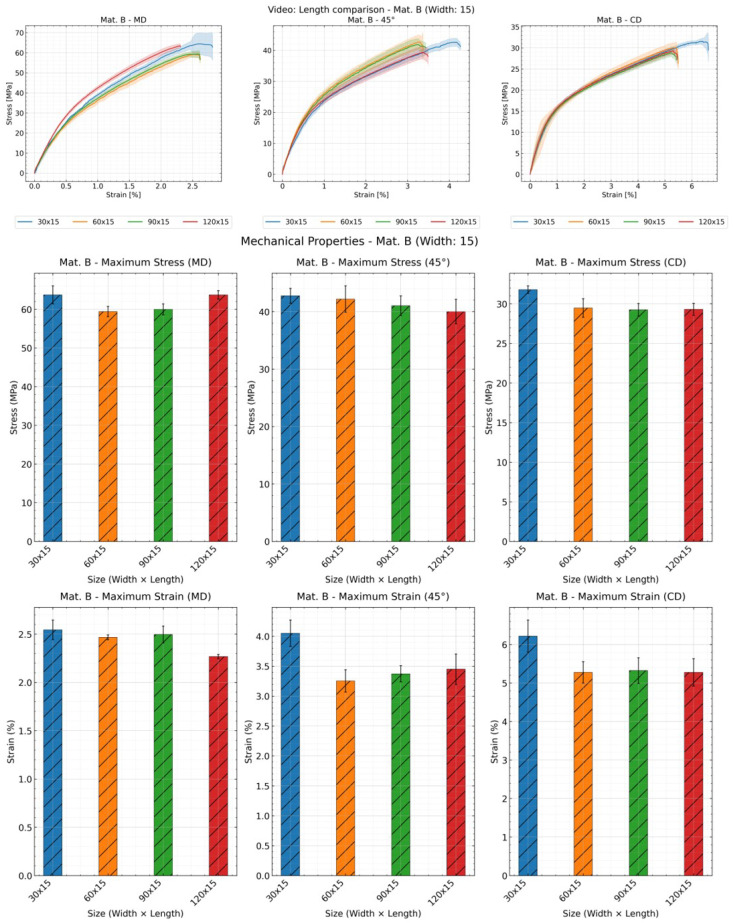
Effect of specimen length on the mechanical properties: stress–strain curve, tensile strength and strain at break of material B with sample width of 15 mm and all lengths using video extensometer; shaded areas represent deviation.

**Figure 7:**
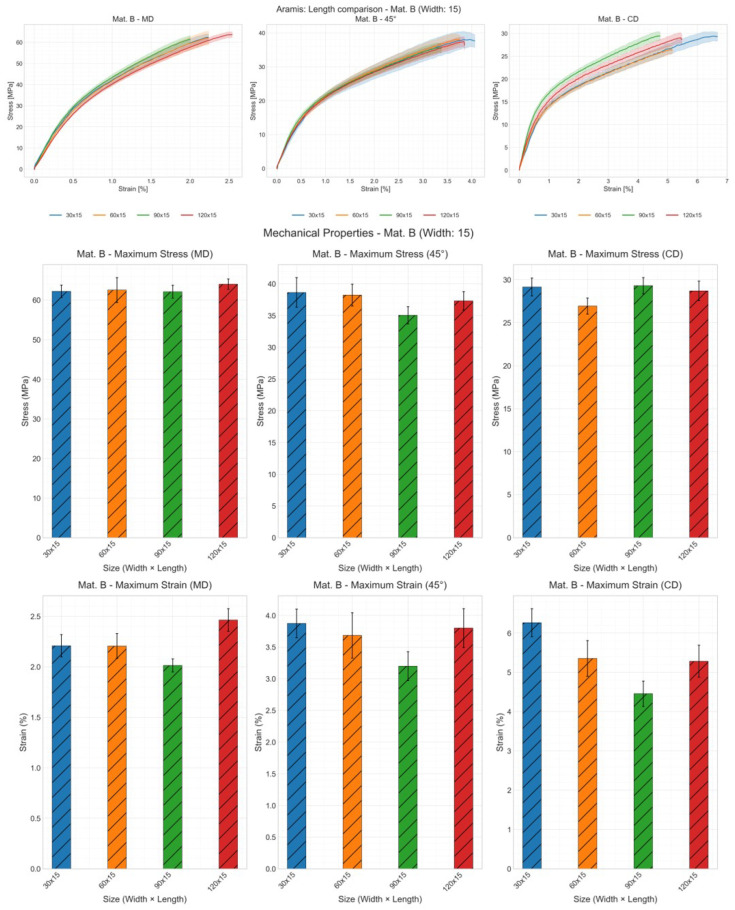
Effect of specimen length on the mechanical properties: stress–strain curve, tensile strength and strain at break of material B with sample width of 15 mm and all lengths using DIC; shaded areas represent deviation.

Figure 5, Figure 6, and Figure 7 showed the influence of the specimen length using material B in three directions, with sample width of 15 mm as an example, using traverse, video extensometer, and digital image correlation (DIC), respectively. Samples with a length of 30 mm exhibited greater deviation than other samples, indicating poorer reproducibility for short specimens. Using the traverse method, a weakly correlated result was observed, which was inconsistent with results obtained using other methods. However, in most cases, the curve of the sample with dimensions of 30 × 15 mm^2^ was clearly longer than the other three dimensions.

Although the stress–strain curve fully represents the material’s mechanical properties, it is difficult to discern the fracture data due to overlap from repeats. Therefore, tensile stress and strain at break were also presented for comparison. As grip length increases, tensile strength tends to decrease, though not always, while fracture strain generally increased (except for the 120 mm length using DIC). Notably, under the traverse method (ref. Figure 5), the trend of maximum stress variation with length is nearly linear. In contrast, only specimens with a length of 30 mm exhibit significantly higher strain values at fracture under other methods. This is because the traverse method cannot distinguish between slip, fixture deformation, and specimen deformation. The amount of slip correlates with measurement time, and at a constant speed, it is positively correlated with the clamping length. Thus, the relationship between specimen length and maximum strain is linear under traverse testing. However, video extensometers and DIC can distinguish slip from specimen deformation; therefore, they do not exhibit linear relationships. Under the DIC method, the maximum strain for the 120 mm specimen is markedly higher than that measured by other methods (ref. Figure 7). Longer specimens provided a greater measurement length within which heterogeneous elongation and deformation can be detected more effectively. In DIC, surface deformation is measured across larger regions within the image field. This allows for the detection of localized deformation clusters, plastic elongation, and contraction—phenomena that are difficult to detect or capture completely on short specimens. This is DIC’s unique advantage for specimens exhibiting non-uniform deformation, particularly those with significant localized deformation.

**Figure 8:**
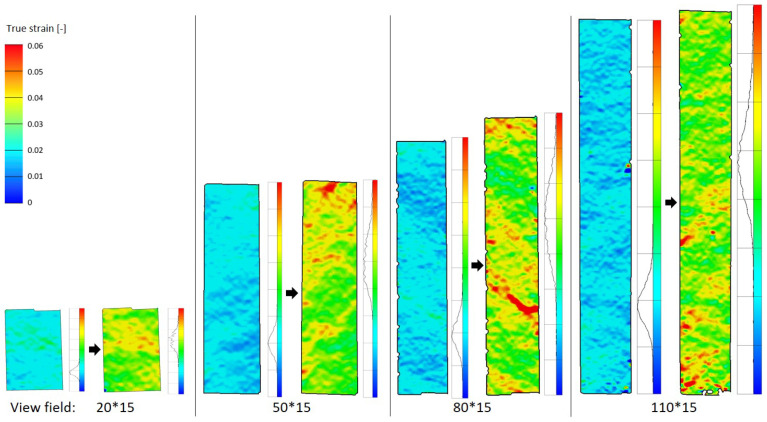
Ful field view of strain distribution from DIC (B-45°): 40% elongation at break and before break.

As the paper material is heterogeneous, it is meaningful to compare the local strain of specimens with different clamping lengths. Since the area close to the clamp is subject to measurement noise, the evaluation length is 10 mm shorter than the clamping length. As the length increases, the specimen exhibits a greater number of “weak points” (especially red area in Figure 8 in 45° direction). At 40% elongation at break, it exhibits more uniform deformation, but weak points are already discernible. During the latter stages, longer specimens of identical width but varying lengths exhibit a larger total area of high-strain regions, thereby increasing the possibility of fracture. The stress pattern of the 45° specimen also exhibits a diagonal pattern, and the final fracture angle is also about 45°.

#### 3.2.2. Effect of Specimen Width

**Figure 9:**
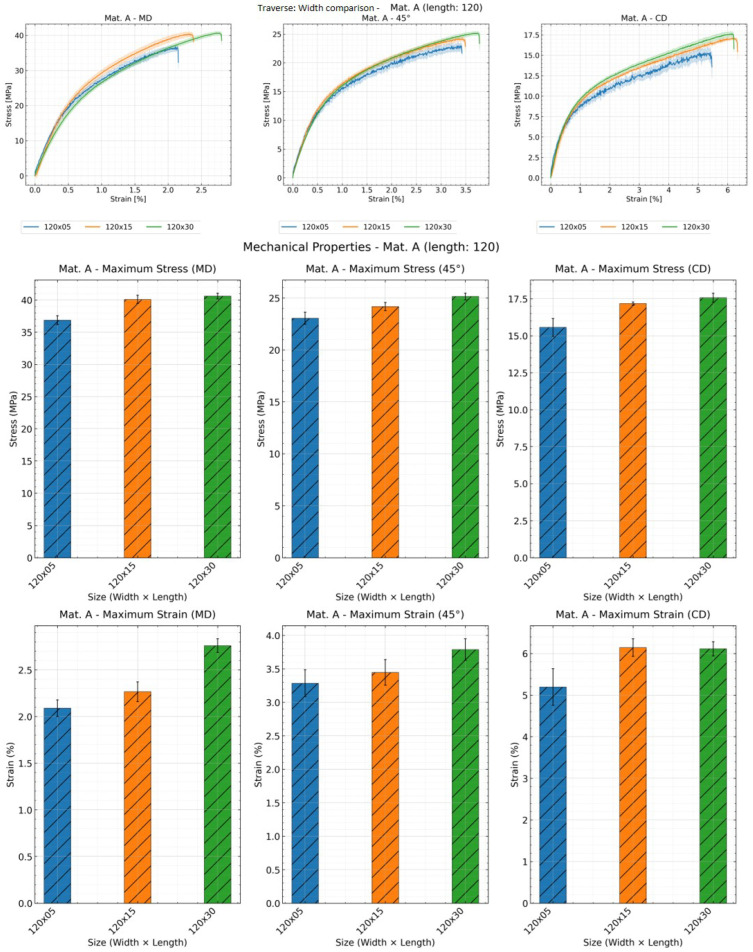
Effect of specimen width on the mechanical properties: stress–strain curve, tensile strength and strain at break of material A with sample length of 120 mm and all widths using traverse; shaded areas represent deviation.

**Figure 10:**
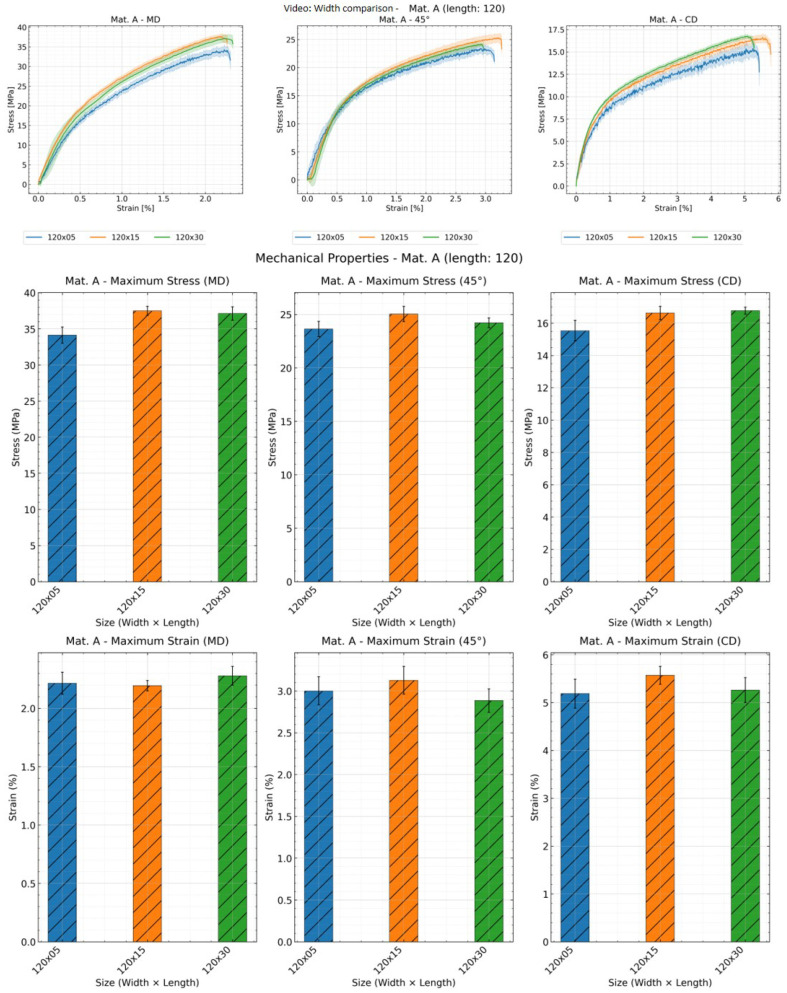
Effect of specimen width on the mechanical properties: stress–strain curve, tensile strength and strain at break of material A with sample length of 120 mm and all widths using video extensometer; shaded areas represent deviation.

**Figure 11:**
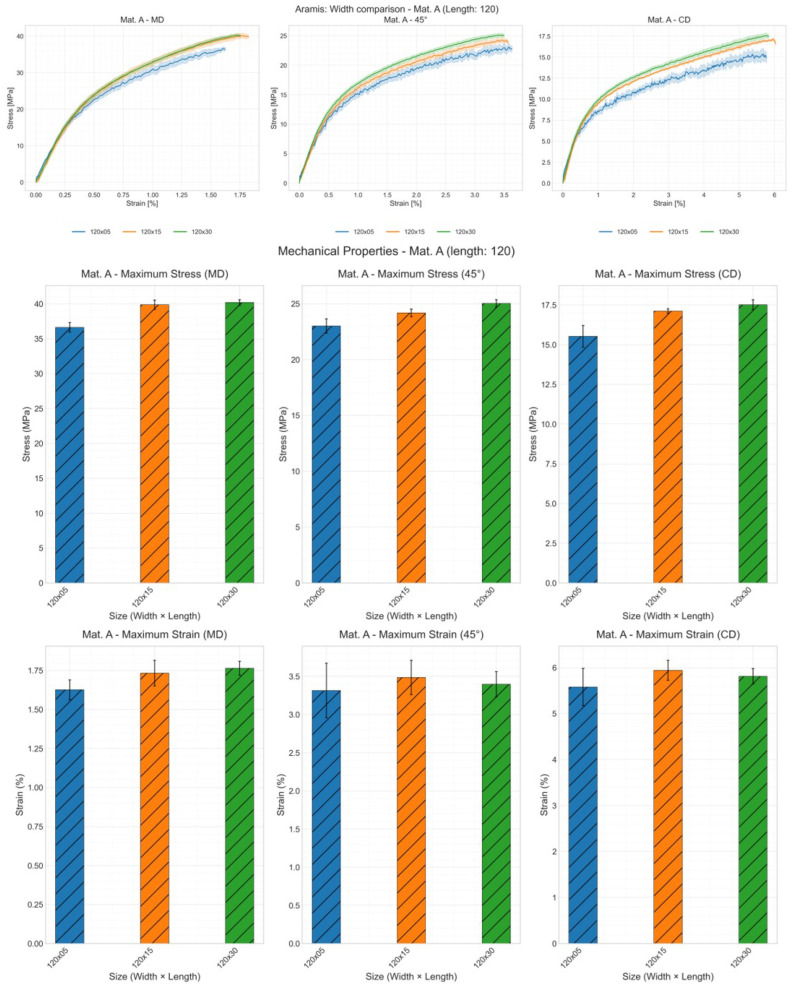
Effect of specimen width on the mechanical properties: stress–strain curve, tensile strength and strain at break of material A in CD with sample length of 120 mm and all widths using DIC; shaded areas represent deviation.

As illustrated in Figure 9, Figure 10 and Figure 11, the influence of width on the stress–strain curve can only be observed through DIC measurements. Both tensile strength and elongation at break increase with width, though there are some exceptions. Analysis of stress–strain curve results reveals that samples with a width of 5 mm exhibit greater deviation across all three methods, indicating poorer reproducibility for narrow specimens.

**Figure 12:**
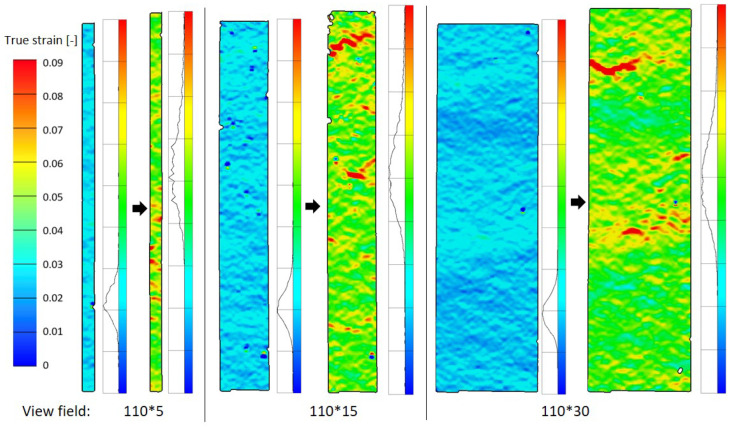
Full field view of strain distribution from DIC (A-CD): 40% elongation at break and before break.

An examination of the full field of local true strain distributions reveals that weak points typically occur at the edge of the specimen rather than in the central region (ref. Figure 12 in CD). The proportion of the edge area relative to the total specimen area depends on width, so narrower specimens are more likely to fracture earlier. However, the specific weak points also depend on the specimen’s inherent inhomogeneity—that is, areas of lower density typically exhibit a higher probability of red strain occurrence [25].

### 3.3. Comparison Under Identical Strain Status from DIC

**Figure 13:**
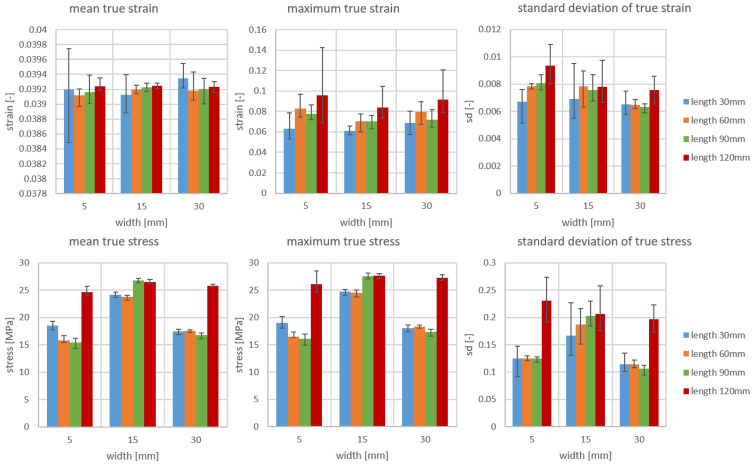
Comparison of mean, maximum, and standard deviation of true strain and true stress at 4% mechanical strain of material B, CD.

**Figure 14:**
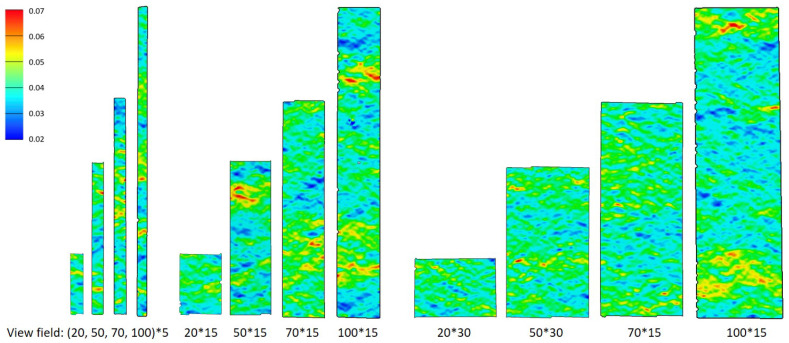
Full field view of strain distribution at 4% mechanical strain of material B, CD.

To better understand how size influences material response in tension tests, several parameters were analyzed under the same average mechanical strain of strain field, including the mean, maximum, and standard deviation of true strain and true stress. Using the CD of material B as an example, the comparison results and visualization results are shown in Figure 13 and Figure 14, respectively. The deviation is derived from five repeated experiments.

In most cases, the mean, maximum value, and standard deviation of true strain all increase with increasing length. As shown in the maximum strain chart, the maximum stress of the red sample (with a 120 mm clamping length) is twice that of the blue sample (with a 30 mm clamping length). Since significant stress concentration is the primary cause of specimen fracture, fracture typically occurs earlier as length increases. However, as the figure shows, length does not always influence fracture behavior. Furthermore, regardless of geometric dimensions, the average stress in specimens varies little, meaning that stress–strain curves are unaffected by size. However, width affects the standard deviation of true strain linearly; therefore, as width increases, the standard deviation decreases. This is a reasonable phenomenon because measurement noise affects the standard deviation, and noise typically occurs at the edges of the sample. Therefore, as width increases, the proportion of noise decreases, resulting in a slight reduction in standard deviation.

For true stress, no other effects of length could be detected beyond the fact that the mean, maximum value, and standard deviation were higher for the 120 mm-long specimens than for specimens of other lengths. No monotonic effect was observed for width either. It is important to note that stress values are particularly pronounced in 15-mm-wide specimens (except when the length is 120 mm), exceeding those of specimens with widths of 5 mm and 30 mm. This phenomenon may stem from the combined effects of edge effects and stress concentration. Extremely narrow specimens (5 mm) are more susceptible to edge defects, notch effects, or clamping stresses, potentially leading to premature failure and reduced measured stress values. Overly wide specimens (e.g., 30 mm) may harbor more defects or inhomogeneities (such as fiber misalignment or weak points), thereby reducing the average strength. Compared to the true strain results, the maximum and average values of true stress are quite close, which is due to the fact that the calculation of true stress is based on the following equation:

(1) $$\begin{array}{c} \sigma_{t}=\sigma \cdot \exp\left(\epsilon_{t}\right)\\ \epsilon_{t}=\ln\left(1+\epsilon \right) \end{array}$$

where σ and ϵ are engineering stress and engineering strain, $\sigma_{t}$ and $\epsilon_{t}$ are true stress and true strain. Engineering strain is derived from image analysis, enabling the calculation of true stress. Engineering stress, on the other hand, remains constant for the field components of the same sample. With very small elongation, the influence of $\exp\left(\epsilon_{t}\right)$ is minimal, resulting in a small discrepancy.

### 3.4. Effect of Sample Area and Length Width Ratio

**Figure 15:**
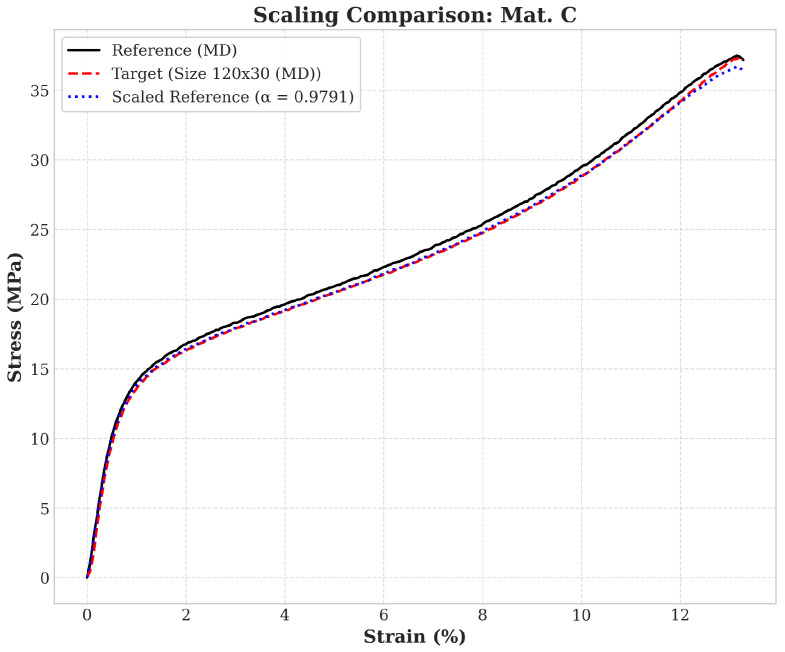
Example of calculation the scaling factor according to reference.

To quantitatively compare the influence of sample geometry on mechanical behavior, a scaling factor α is introduced to characterize the stress–strain curve relative to a reference curve. Using full-field analysis in the DIC method, a reference sample measuring 90 mm in length and 30 mm in width (i.e., α=1) is employed. Taking a sample of material C oriented in the MD direction, measuring 120 mm in length and 30 mm in width, the calculation of the scaling factor is illustrated as shown in Figure 15.

**Figure 16:**
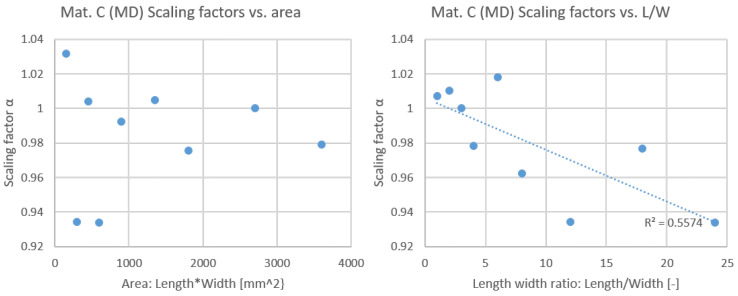
Scaling factors vs. sample area and vs. length-to-width ratio of material C in MD.

Two charts (Figure 16) were plotted for scaling factors versus sample area (i.e., the product of length and width) and versus length-to-width ratio (i.e., length divided by width). The surface area of the sample is not related to the scaling factor. The surface area of the sample is not correlated with the scaling factor, but the length-to-width ratio exhibits a weak correlation with the scaling factor, with an R-squared value of 0.5574. The authors do not believe that such R-squared values can be considered indicative of a size effect in paper and paperboard.

**Figure 17:**
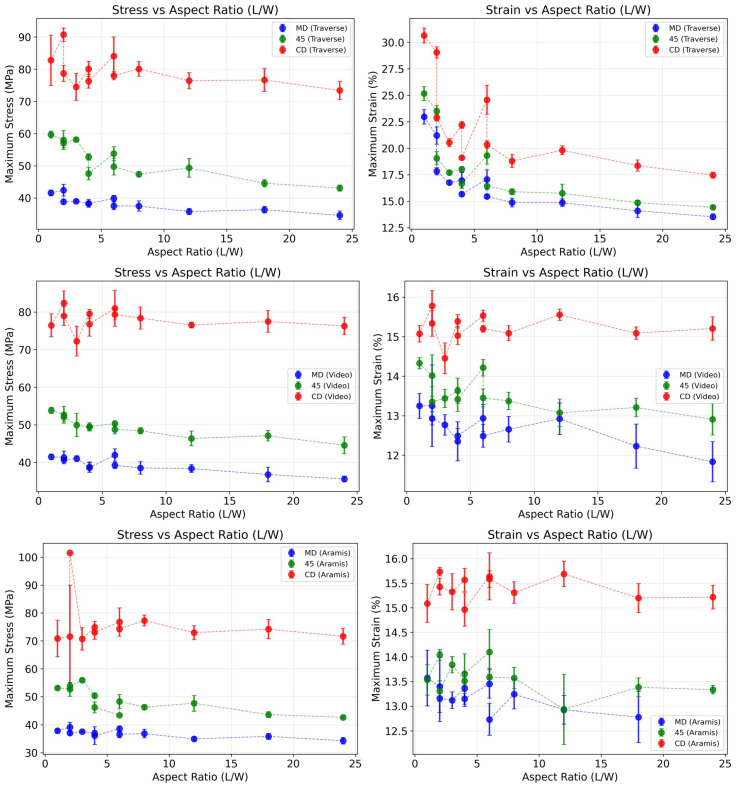
Maximum stress and maximum strain vs. length, width, and length-to-width ratio of material C in three directions.

To better compare the effects of aspect ratio on mechanical properties, Figure 17 illustrates how maximum stress and strain influence three directions of paperboard C under three measurement methods (from top to bottom: traverse, video extensometer, and DIC). A clear correlation is observable in Traverse measurements but not in the other two methods. This is related to the traverse measurement principle, which records not only specimen deformation, but also factors such as slippage, fixture deformation, and internal stress effects.

## 4. Discussion

Some correlations can be observed in the results, but not all experiments follow the assumed relationship between specimen size and mechanical properties. In particular, the correlation with stress–strain curves is weak and is limited to measurement using the traverse strain method. The authors argue that the hypothesized geometric correlation does not exist during tensile testing and attribute experimental variations to external factors. Because paper specimens are thin, securing them for testing is difficult and invariably results in some degree of slip, a phenomenon verifiable via DIC method. Since longer specimens exhibit significantly more slippage than shorter ones during testing, a substantial portion of the deviation observed in traverse measurements for different specimen lengths stems from this slippage. Additionally, short specimens are prone to bending deformation during clamping, generating preload in unloaded regions and exacerbating material fracture strain. Machine stiffness also influences deformation during testing and will be included in the traverser recording. Therefore, this paper recommended using DIC or a video extensometer to record deformation during tensile testing and making manual adjustments as necessary to prevent preload from influencing the results. To ensure data accuracy, avoid using specimens that are excessively thin (width < 5 mm) or short (length < 30 mm). To minimize the impact of non-edge fibers on the results, the samples must be cut with a sharp blade. A fixture designed to reduce slippage, more suitable for paper and paperboard materials, should also be developed. Furthermore, due to the inhomogeneous nature of paperboard materials and their thin layers, more repetition tests are recommended, as their stress–strain curves exhibit greater deviation than those of other materials.

The dependence of strength on dimensions in engineering mechanics, known as the size effect, is explained by classical theories of elastic or plastic structures. In larger or longer specimens, such as a rope or concrete cube, flaws are more likely to occur, leading to a decrease in strength [26]. This effect is also seen in paperboard specimens, which may fracture earlier, resulting in a shorter stress–strain curve. For larger cross-sections, local weak points are less problematic than for smaller ones, resulting in increased strength. However, the paperboard materials used consisted of short fibers, and the fiber length was not on the same scale as the specimen width. Thus, the width factor did not significantly impact the results, but it introduced measuring noise in the DIC method. Clearly, sample size significantly affects fracture behavior compared to the stress–strain curve, particularly strain at the point of rupture. Therefore, a size-dependent fracture model is worthwhile for fibrous materials.

## 5. Conclusions

Tensile tests utilizing three distinct strain measurement mechanisms (traverse, video extensometer, and digital image correlation (DIC)) demonstrate poor correlation between sample size and the stress–strain curve, though sample size affects fracture. This study compared these three measurement mechanisms and concluded that the digital image correlation (DIC) method and video extensometer are superior for tensile testing of paper materials due to their ability to avoid the influence of sample slip at the clamps. The DIC method can also measure the deformation of the entire specimen surface with greater accuracy and provide local strain information. Absolute size effects are absent; however, specimens that are too thin or too short should be avoided due to the possibility that other factors, such as slipping at the clamp or internal pre-stress, will affect the results. Additionally, specimen geometry primarily influences the fracture properties—specifically tensile strength and strain at break—rather than the fundamental constitutive behavior of the stress–strain curve.

## Figures and Tables

**Table 1:** Information on the paper materials used in the work.

Material	Grammage [g/m^2^]	Thickness [mm]	MD-CD Ratio [-]
A	250	0.29	2.42
B	310	0.42	2.75
C	310	0.35	0.95

**Table 2:** Comparison of measurement results using three different test setups.

Mat.-Dir.	Stress–Strain Curve						Maximum Stress						Maximum Strain						Sum	
Methods	TRA		VID		DIC		TRA		VID		DIC		TRA		VID		DIC		All	
	L	W	L	W	L	W	L	W	L	W	L	W	L	W	L	W	L	W	L	W
A-MD	0	0	0	0	1	0	1	1	0	0	1	1	1	1	0	1	0	0	4	4
A-45°	0	0	1	0	0	0	1	0	1	0	1	0	1	1	0	1	0	1	5	3
A-CD	1	0	0	0	1	0	1	1	1	1	1	0	0	1	0	1	0	1	5	5
B-MD	0	0	0	0	0	0	1	0	0	1	1	0	1	1	1	0	0	0	4	2
B-45°	0	1	0	0	0	0	0	0	1	0	0	1	1	1	0	0	0	1	2	4
B-CD	1	0	0	0	0	0	0	1	1	0	0	1	1	1	1	1	0	1	4	5
C-MD	0	1	0	0	0	0	1	1	1	1	1	1	1	1	1	1	0	1	5	7
C-45°	0	0	0	0	0	1	1	1	1	1	1	1	1	1	1	1	0	1	5	7
C-CD	1	1	0	0	0	0	0	0	1	0	0	0	1	1	1	0	0	0	4	2
Sum	3	3	1	0	2	1	6	5	7	4	6	5	8	9	5	6	0	6	38	39

Number Meaning: 1 = clear correlation (Fully compliant when considering deviations), 0 = weak correlation; Abbreviations: Mat. = Material, Dir. = Direction, L = Length (left column), W = Width (right column).

## Data Availability

The original contributions presented in this study are included in the article. Further inquiries can be directed to the corresponding authors.

## References

[1] Niskanen K. (2008). Paper Physics.

[2] Cox H.L. (1952). The elasticity and strength of paper and other fibrous materials. Br. J. Appl. Phys..

[3] Wahlström T. (2009). Prediction of Fibre Orientation and Stiffness Distributions in Paper—An Engineering Approach. Proceedings of the 13th Fundamental Research Symposium.

[4] Sanjon C.W., Leng Y., Hauptmann M., Groche P., Majschak J.P. (2024). Methods for Characterization and Continuum Modeling of Inhomogeneous Properties of Paper and Paperboard Materials: A Review. BioResources.

[5] Sanjon C.W., Leng Y., Hauptmann M., Groche P., Majschak J.P. (2024). Transmitted Light Measurement to Determine the Local Structural Characteristics of Paperboard: Grammage, Thickness, and Fiber Orientation. Fibers.

[6] Marin G., Nygårds M., Östlund S. (2020). Elastic-plastic model for the mechanical properties of paperboard as a function of moisture. Nord. Pulp Pap. Res. J..

[7] Yoshihara H., Yoshinobu M. (2014). Effects of specimen configuration and measurement method of strain on the characterization of tensile properties of paper. J. Wood Sci..

[8] Vial G. (2004). Video extensometers. Adv. Mater. Process..

[9] Choi D., Thorpe J., Hanna R. (1991). Image analysis to measure strain in wood and paper. Wood Sci. Technol..

[10] Considine J., Scott C., Gleisner R., Zhu J. (2005). Use of digital image correlation to study the local deformation field of paper and paperboard. Proceedings of the 13th Fundamental Research Symposium Conference.

[11] Considine J.M., Pierron F., Turner K.T., Vahey D.W. (2014). Anisotropy Evaluation of Paperboard with Virtual Fields Method. Residual Stress, Thermomechanics & Infrared Imaging, Hybrid Techniques and Inverse Problems.

[12] Considine J.M., Pierron F., Turner K.T., Lava P., Tang X. (2017). Smoothly varying in-plane stiffness heterogeneity evaluated under uniaxial tensile stress. Strain.

[13] (2008). Paper and Board—Determination of Tensile Properties.

[14] (2006). Tensile Properties of Paper and Paperboard (Using Constant Rate of Elongation Apparatus).

[15] (2022). Standard Test Method for Tensile Properties of Paper and Paperboard Using Constant-Rate-of-Elongation Apparatus.

[16] Lahti J.A., Dauer M., Keller D.S., Hirn U. (2020). Identifying the weak spots in packaging paper: Local variations in grammage, fiber orientation and density and the resulting local strain and failure under load. Cellulose.

[17] Lahti J., Dauer M., Keller D.S., Hirn U., Söderberg D. (2017). Linking Paper Structure to Local Distribution of Deformation and Damage. Proceedings of the Progress in Paper Physics Seminar.

[18] Na Y., Muhlstein C. (2019). Relating Nonuniform Deformations to Fracture in Uniaxially Loaded Non-Woven Fiber Networks. Exp. Mech..

[19] Malmberg B. (1964). Remslängd och töjningshastighet vid spänningtöjningsmätningar på papper. Sven. Papp..

[20] Tryding J. (1996). In-Plane Fracture of Paper.

[21] Yokoyama T., Nakai K., Odamura T. (2007). Tensile stress-strain properties of paper and paperboard and their constitutive equations. J. Jpn. Soc. Exp. Mech..

[22] Hagman A., Nygårds M. (2012). Investigation of sample-size effects on in-plane tensile testing of paperboard. Nord. Pulp Pap. Res. J..

[23] Dolatshahi S., Kortschot M.T. (2009). The Effect of Fibre Orientation on the Zero-Span Testing of Paper. Proceedings of the 13th Fundamental Research Symposium.

[24] Johansson S., Engqvist J., Tryding J., Hall S.A. (2021). 3D Strain Field Evolution and Failure Mechanisms in Anisotropic Paperboard. Exp. Mech..

[25] Krasnoshlyk V., Du Roscoat S.R., Dumont P.J., Isaksson P. (2018). Influence of the local mass density variation on the fracture behavior of fiber network materials. Int. J. Solids Struct..

[26] Bažant Z.P. (1984). Size effect in blunt fracture: Concrete, rock, metal. J. Eng. Mech..

